# Crotonylation modification and its role in diseases

**DOI:** 10.3389/fmolb.2024.1492212

**Published:** 2024-10-31

**Authors:** Yi Guo, Junqin Li, Kaiming Zhang

**Affiliations:** Shanxi Key Laboratory of Stem Cell for Immunological Dermatosis, Institute of Dermatology, Taiyuan City Central Hospital of Shanxi Medical University, Taiyuan, China

**Keywords:** post-translational modification, crotonylation, histone, non-histone protein, disease

## Abstract

Protein lysine crotonylation is a novel acylation modification discovered in 2011, which plays a key role in the regulation of various biological processes. Thousands of crotonylation sites have been identified in histone and non-histone proteins over the past decades. Crotonylation is conserved and is regulated by a series of enzymes including “writer”, “eraser”, and “reader”. In recent years, crotonylation has received extensive attention due to its breakthrough progress in reproduction, development and pathogenesis of diseases. Here we brief the crotonylation-related enzyme systems, biological functions, and diseases caused by abnormal crotonylation, which provide new ideas for developing disease intervention and treatment regimens.

## 1 Introduction

Post-translational modifications (PTMs) of proteins significantly impact their biological functions, including gene expression, cell growth, embryonic development, metabolism and other biological processes (Zhao et al., 2018). Crotonylation is one of lysine acylation modification that transfers the crotonyl group onto lysine residues by using crotonyl-CoA as substrate under the action of crotonyltransferase (Tan et al., 2011). To date, multiple lysine crotonylation sites of histones and non-histones have been identified in various organisms (Wei et al., 2017a; Xu et al., 2017; Sun et al., 2017; Kwon et al., 2018; Liu et al., 2018; Lu et al., 2018).

Although the regulatory enzyme systems and targets of crotonylation partially overlap with those of acetylation, the peculiar CC bond structure of crotonylation suggests that crotonylation may have specific biological functions (Tan et al., 2011). The hydrophobic groups of crotonylation neutralize the positive charge of lysine residues, which influences the function of substrate proteins. Lysine crotonylation modification is involved in a variety of key cellular processes, such as nucleotide metabolism, chromatin recombination, enzyme activity, and regulation of protein activity and protein localization, thereby mediating multiple physiological and pathological processes of the body. More importantly, crotonylation of protein could also affect the development and progress of diseases, such as cancer, tissue injury, neuropsychiatric disease, and virus infection. In this review, we summarize the writers, erasers and readers of lysine crotonylation, and their physiological functions. In addition, we further discussed the functional importance of crotonylation modification in the pathogenesis of various diseases, which may provide new insights into the epigenetic research of related diseases.

## 2 Post-translational modification (PTM) of proteins

The living organism is a complex and dynamic system, which is constantly metabolized to produce new substances, remove waste and harmful substances, and coordinate the functions of various parts. Disturbance of this system will cause abnormal functions and the development of the diseases. Proteins are the executors of various functions, such as immunity, apoptosis, signal transduction, stimulus response and individual development, in the body. Thus, the function of organism is entirely determined by the functions of proteins.

PTM of proteins is an important way of change in protein structure, regulating protein function. It is estimated that 50%–90% of proteins in the human body undergo PTM, including splicing of peptide chain backbones, adding new groups to specific amino acid side chains, and chemical modification of existing groups (Doyle and Mamula, 2001; Zhao and Jensen, 2009). Many physiological functions of cells, including cellular response to the external environment, are achieved through dynamic PTM of proteins. At present, more than 400 PTMs have been identified, and the common modification processes include phosphorylation, ubiquitination, methylation, acetylation, glycosylation, nitrosylation, oxidation, etc. (Berstein et al., 2007; Bannister and Kouzarides, 2011). However, the protein modification process we have mastered is still very limited, with a larger portion (at least 70%) of unknown modification, including unknown modification types, modification proteins and modification sites. The PTM of protein lysine is one of the important regulatory mechanisms (Chen et al., 2007; Xie et al., 2012; Zhang et al., 2011). Epigenetic regulation is involved in almost all life activities, such as gene expression, cell proliferation, RNA splicing and editing, embryonic development and other important biological processes. Alteration in epigenetic regulation can affect the whole life activities, possibly resulting in the development of diseases (Chi et al., 2010; Laird and Jaenisch, 1996; Jiang et al., 2004).

## 3 Crotonylation modification

### 3.1 Histone crotonylation modification

**FIGURE 1:**
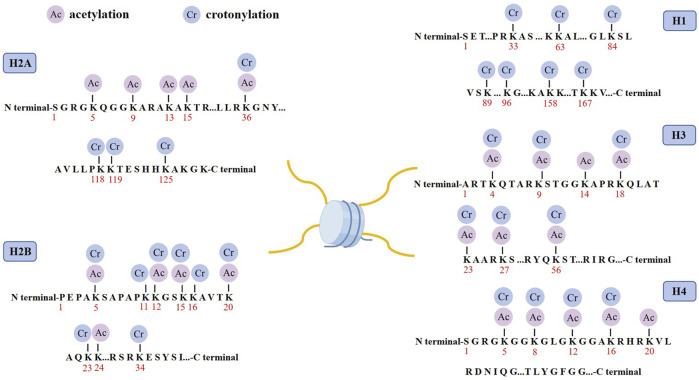
Distribution of crotonylation and acetylation on the histones. Schematic model shows the principal lysine crotonylation sites and acetylation sites on histones.

**FIGURE 2:**
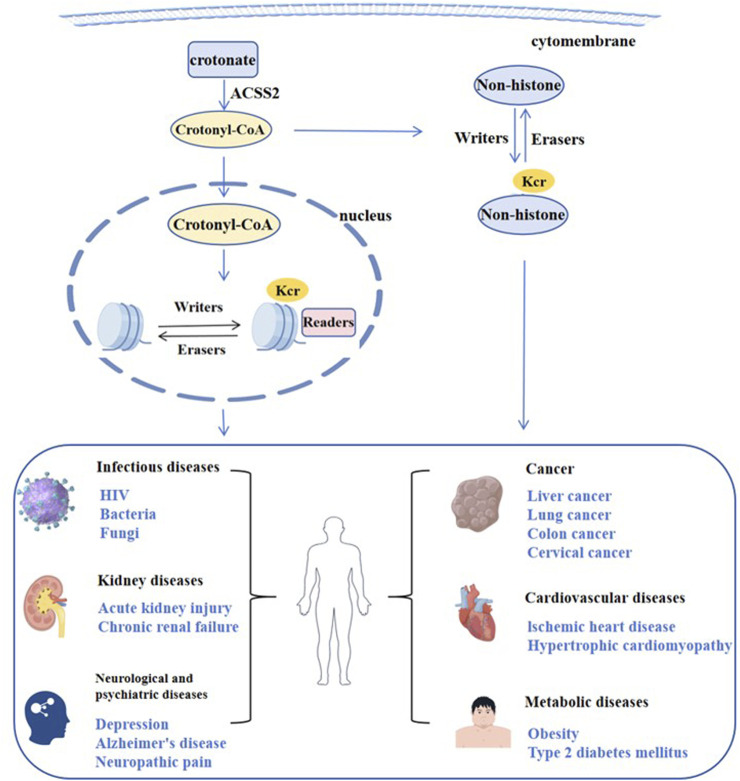
Regulation of crotonylation and related diseases.

Histone crotonylation is an evolutionarily conserved histone PTM, widely present in eukaryotic cells and enriched in the transcriptional initiation region, suggesting that histone crotonylation may be involved in transcriptional regulation. Crotonylation modification mostly occurs on lysine, in which crotonyl group is transferred to lysine residue with crotonyl coenzyme A (Cr-coA) as substrate under the action of histone crotonyltransferase. Crotonyl group is an organic compounds with specific structure that can affect its interaction with biomolecules. Structurally, the histone crotonyl group is very similar to the acetyl group, with only one more carbon-carbon double bond, thus resulting in a unique rigid planar conformation (Sabari et al., 2017). Except the similarity in structure, there is also an overlap between crotonylation and acetylation modifications at the histone sites (Figure 1), indicating that part of the same biological function may exist between the two modifications. The formation process of crotonylation is similar to other acylation modification, which is not only affected by the concentration of crotonyl-CoA in cells, but also regulated by a series of enzymes (Figure 2). The regulatory factors of crotonylation are shown in Table 1.

**TABLE 1:** The regulatory factors for crotonylation.

Regulatory pattern	Factor	Crotonylation site
Writer	CBP/p300	H3K18
	MOF	H3K4, H3K9, H3K18, H3K23, H4K8, H4K12
	Esa1	H4K5, H4K8, H4K12, H4K16
	GCN5	H3K9, H3K14, H3K18, H3K23, H3K27
	Hat1, Rtt109	H3K9
	HBO1	H3K14, H4K12
Eraser	SIRT 1–7	H3K4, H3K9, H3K18, H3K27, H4K8
	HDAC1, 2, 3, 8	H3K4, H3K9, H3K18, H3K23, H4K8, H4K12
	FOSIR5	H3K18
Reader	TAF14	H3K9
	YEATS2	H3K27
	AF9	H3K9, H3K18, H3K27
	MOZ, DPF2	H3K14
	GAS41	H3K27

#### 3.1.1 The crotonyltransferase of crotonylation (Writer)

Histone crotonyltransferase (HCT) uses Cr-CoA as a substrate to transfer crotonyl groups to lysine residues and promotes the histone crotonylation modification. Given that histone acetyltransferase (HAT) has widely acyltransferase activity (Lee and Workman, 2007; Roth et al., 2001), most of the enzymes that catalyze histone crotonylation are currently sought from HAT. CBP is the binding protein of cAMP response element-binding (CREB) protein, while p300 is adenovirus protein ElA-binding protein, with a molecule weight of ≈300KD. Because of their structural similarity and functional redundancy, they are often called together as CBP/p300 (Chakravarti et al., 1996). P300/CBP has both HAT and HCT activities (Sabari et al., 2015; Kaczmarska et al., 2017). Histone crotonylation modification is influenced by the concentration of intracellular Cr-CoA complexes. Compared with catalytic histone acetylation, p300/CBP catalyzed histone crotonylation can stimulate transcription more effectively (Huang et al., 2018). P300/CBP is the predominant HCT in mammalian cells, which can promote transcription by increasing histone crotonylation modification and recruiting AF9/DPF2 (Wei et al., 2017b).

In addition to p300/CBP, MOF (Males absent on the first) also exhibits crotonyltransferase activity (Liu et al., 2017). MOF is an evolutionarily conserved crotonyltransferase. Human MOF is very similar to *Drosophila* MOF, both containing HAT enzyme activity domain, chromo domain and C2H2 zinc-finger domain. MOF can catalyze histone crotonylation at multiple sites of H3 and H4. Esa1, a homologous to MOF in yeast, also catalyzes histone crotonylation. H3K9cr levels are abolished or reduced considerably in the yeast HAT (HAT1, Gcn5, and Rtt109) deletion strains. However, neither human MOF nor yeast Esa1 or yeast Gcn5 displays any HCT activity *in vitro*, suggesting they may exert HCT activity by forming complex (Decker et al., 2008; Smith et al., 1998; Kollenstart et al., 2019).

HBO1 is a versatile histone acyltransferase that catalyzes not only histone acetylation but also propionylation, butyrylation and crotonylation both *in vivo* and *in vitro*. It was found that HBO1 is required for H3K14cr and contributes to H4K12cr, actransient knockdown of HBO1 in cells results in substantial reduction of histone crotonylation (Xiao et al., 2021). It is noteworthy that knockout or knockdown of HBO1 appears to affect specifically histone acylations, as HBO1 knockout or knockdown had no obvious effect on non-histone protein acylation.

#### 3.1.2 The decrotonylase of crotonylation (Eraser)

Like histone acetylation modification, histone crotonylation modification is also a dynamic equilibrium process *in vivo*. Histone acetyltransferases can catalyze histone crotonylation, and histone deacetylase can also remove the histone crotonylation. Histone deacetylases (HDACs) are mainly divided into four categories: Class I, Class II, Class III and Class IV (Seto and Yoshida, 2014). Class I includes HDAC1, HDAC2, HDAC3 and HDAC8, while Class III includes Sirt1-Sirt7. Sirt1, Sirt2 and Sirt3 have histone decrotonylation activity (Feldman et al., 2013), and Sirt3 can regulate the transcription of corresponding genes by regulating the crotonylation level of histone H3K4 (Bao et al., 2014). Class I (HDAC1∼3, HDAC8) also has histone decrotonylase activity (Madsen and Olsen, 2012), and selective removal of histone crotonylation can extensively inhibit transcription. HDAC-VRPP mutant, which specifically remove histone crotonacylation modification but not histone acetylation modification, can still inhibit transcription. In addition, it has been demonstrated that FoSir5 (an ortholog of the human lysine deacetylase SIRT5) can modulate ATP synthesis through lysine decrotonylation in different organelles and thus conidial germination of *Fusarium oxysporum* (Zhang et al., 2021). Histone crotonylation modification has unique biological functions, but it is unknown whether there are other enzymes besides histone acetyltransferase or histone deacetylase are involved in histone crotonylation.

#### 3.1.3 The readers of crotonylation

Specific effector proteins recognize histone crotonylation modification, which is a prerequisite for further function. YEATS, Bromodomain and Double PHD finger (DPF) are three classes of domains that recognize acylation (Jenuwein and Allis, 2001). The second Bromodomain of TAF1 can recognize crotonylation and butyrylation (Flynn et al., 2015), while other Bromodomains cannot recognize histone crotonylation modification. Due to the unique carbon-carbon double bond structure of crotonylation modification, YEATS can recognize crotonyl groups through its aromatic hydrocarbon structure, and its ability to bind to histone crotonylation is 2–4 times of histone acetylation (Andrews et al., 2016). The YEATS structural domain of AF9 can specifically recognize the histone crotonylation modification site H3K9, thus promoting transcription (Li et al., 2016; Zhang et al., 2016; Jiang et al., 2020). The YEATS domains in YEATS2 and Taf14 have better crotonyl binding ability, which can specifically recognize the crotonylation of H3K27 and H3K9 sites, regulating gene expression (Dan et al., 2016; Andrews et al., 2016).

GAS41 (a member of the human YEATS domain family) is involved in epigenetic signaling primarily as a reader recognizing histone modification, including histone acetylation, benzoylation, succinylation and crotonylation. By binding to H3K27cr, GAS41 can be recruited by transcription factor MYC to the SIN3A-HDAC1 co-repressor to repress the transcription of p21-related genes (Liu et al., 2023). The double PHD zinc-finger structure in MOZ and DPF2 can specifically recognize crotonylation, with a strong binding ability to crotonylation at the histone H3K14 site (Xiong et al., 2016). The zinc-finger structure of PHD recognizes crotonyl through its unique hydrophobic groups and hydrogen bonds, and its ability to bind to crotonyl group is 4–8 times of acetyl group. Because of the unique structure of crotonyl, it is believed that its recognition protein is not only limited to the acylated reader.

#### 3.1.4 Biological functions of histone crotonylation

The structures of histone crotonylation and acetylation are similar, but diverse. Although the modification sites of crotonylation and acetylation overlap, they are different (Wei et al., 2017a; Wang et al., 2021). Thus, crotonylation of histone does not only have similar effects on acetylation, but also has its own unique biological functions. Because the regulatory enzymes and effector proteins have not been thoroughly studied, the biological function of histone crotonylation modification has not been fully understood at present. Based on the available evidence, histone crotonylation modification has the following biological functions:

(1) Regulation of transcriptional activity: crotonylation modification is highly abundant on histones and mostly occurs in the transcription initiation region (Tan et al., 2011; Wei et al., 2017a). Similar to acetylation, crotonyl binds to histones to loosen nucleosomes and facilitates transcription. Catalytic histone crotonylation has a stronger transcription-promoting effect than catalytic histone acetylation (Sabari et al., 2015). In addition to affecting the binding of DNA and histones, the better recognition of crotonylation modifications (such as YEATS and DPF domains) may be another factor affecting transcription.
(2) Regulation of sex chromosome active genes: In mouse and human sperm cells, the level of crotonylation modification is higher and is mostly distributed in the transcriptional initiation region or enhancer region. After meiosis, high abundance of crotonylation modifications is concentrated on sex chromosomes to mark testicular specific genes and affect the expression of active genes (Tan et al., 2011).
(3) Kidney protection: Studies have shown that crotonylation modification maintains a high level in the kidney, which can promote the expression of emergency-induced genes and protect the kidney from injury in the mouse model of acute kidney injury (Liu et al., 2019). Injection of crotonate into mice increases the overall crotonylation modification levels and mitigates acute kidney injury.

In addition, histone crotonylation also regulates self-renewal or cell differentiation of stem cells (Kelly et al., 2018; Fang et al., 2021; Fu et al., 2018; Lv et al., 2021), DNA damage and repair (Yu et al., 2020; Abu-Zhayia et al., 2019), and activation of latent HIV (Jiang et al., 2018).

### 3.2 Non-histone crotonylation modification

Besides histone, crotonylation is also present on a variety of non-histone proteins (Xu et al., 2017). Several enzymes that catalyze the opposite processes of non-histone crotonylation and decrotonylation have been identified. The acetyltransferases, CBP, PCAF and MOF, can catalyze the crotonylation of non-histone proteins, while the deacetylases, HDAC1 and HDAC3, are involved in decrotonylation. Non-histone crotonylation modification has been found in 453 proteins, and 558 specific crotonylation modification sites have been identified in human cervical cancer HeLa cell line (Wei et al., 2017a). Crotonylation was originally considered as a specific marker for sex-related genes of histones and was directly associated with gene transcription (Tan et al., 2011). Subsequent studies have revealed that crotonylation is involved in the pathogenesis of a number of diseases, including acute kidney injury, depression and cancer process, via regulating the structure and function of histones (Jiang et al., 2018; Ruiz-Andres et al., 2016; Liu et al., 2019; Berger and Moeller, 2014). In the past few years, mass spectrometry-based proteomics has greatly accelerated the discovery and identification of endogenous crotonylation proteins, and has quantified the relative impact of thousands of crotonylation sites on genetic and metabolic activities. An increasing number of proteomic analyses have demonstrated the high frequency of non-histone crotonylation. These proteins include many key cellular proteins associated with physiology and diseases in eukaryotes, and crotonylation of these non-histone proteins is extensively involved in almost all major biological processes (Wan et al., 2019a; Liu et al., 2020; Wu et al., 2017).

## 4 Crotonylation associated diseases

As a novel post-translational modification, crotonylation has been implicated in a variety of human diseases. In this section, we primarily summarize the confirmed crotonylation in diseases (Table 2), and the importantly crotonylated proteins and their sites.

**TABLE 2:** The diseases associated with crotonylation.

Diseases	Protein crotonylation	Description
Acute kidney injury	Histone	The protective role of crotonylation in AKI is not only by upregulating the levels of PGC-1α and SIRT3 and downregulating CCL2, but also by blocking TWEAK and thus upregulating SIRT3
Kidney fibrosis	Cox4i1, H3K9	Crotonylation affects renal fibrosis by influencing mitochondrial function and cellular senescence in renal tubular epithelial cells
Depression	Histone	CDYL inhibits neuropeptide VGF expression to affect structural synaptic plasticity
Alzheimer’s disease	H3K27	Knockdown of NEAT1 can upregulate the level of H3K27cr and inhibite the transcription of autophagy-related genes
Neuropathic pain	—	Crotonylation induces macrophage activation and inflammatory
HIV lantency	H3K4	ACSS2 promotes histone crotonylation modification of HIV LTR, leading to reactivation of latent HIV.
Prostate cancer	Histone	Histone crotonylation induced by BRD4 inhibitors can inhibit proliferation and migration in prostate cancer cells
Colorectal cancer	ENO1, H3K27	Crotonylation promotes proliferation, migration and invasion of colorectal cancer cells
Cervical cancer	HNRNPA1	Crotonylation of HNRNPA1 affects its protein expression and proliferation of Hela cells
Leukemia	—	Inhibition of YEATS domain decreases the transcription of oncogenes in leukemia
Glioblastoma	Histone	Glioblastoma stem cells reprogram lysine catabolism to promote histone crotonylation, which affects interferon signaling and T cell infiltration
Hepatocellular carcinoma	—	GCDH alters cellular redox state via crotonylation, leading to HCC cell senescence and immune cell infiltration
hypertrophic cardiomyopathy	H3K18, H2BK12	ECHS1 overexpression inhibits crotonylation of H3K18 and H2BK12
Myocardial I/R injury	IDH3a, TPM1	Crotonylation of IDH3a and TPM1 can protect cardiomyocyte from apoptosis by inhibiting BNIP3 -mediated mitosis or cytoskeleton structure rearrangement
Obesity	Non-histone	Non-histone crotonylation modulates white fat browning
Type 2 diabetes mellitus	XPO1, HSPA8	EPO can alter the crotonylation level of XPO1 and HSPA8, and affect cell senescence and glucose metabolism

### 4.1 Kidney diseases

Acute kidney injury (AKI) refers to sudden and sustained declines in renal function, manifested by azotemia, imbalance of water electrolyte and acid-base, and systemic symptoms (Wang et al., 2021). Induction of AKI with folic acid or cisplatin increases levels of protein crotonylation in the kidneys in mice (Ruiz-Andres et al., 2016). Injection of croton acid into the AKI mice upregulates crotonylation modification and expression levels of both mRNA and proteins of kidney protective factor PGC-1α, decrotonylase and SIRT3, while downregulating mRNA levels of chemokine CCL2, suggesting that the protective role of crotonylation modification in kidney damages is via up-regulating the levels of PGC-1α and SIRT3 and down-regulating CCL2. Moreover, treatment of renal tubular cells with crotonic acid can prevent the downregulation of SIRT3 induced by tumor necrosis factor-like weak inducer of apoptosis (TWEAK). TWEAK is a member of the tumor necrosis factor ligand superfamily, which promotes inflammation and AKI, suggesting that increase crotonylation might have a beneficial effect on AKI (Ruiz-Andres et al., 2016).

Recently, studies have revealed the mechanism of neuropilin-1 (Nrp1) regulating renal injury and renal fibrosis. This study found that Nrp1 can activate the TNF-α signaling pathway, enhance the interaction of the transcription factor Nfkb1 and Etv6, and inhibit the transcription of Etv6, while the transcription factor Etv6 can positively regulate the expression of acyl-CoA oxidase 3 (Acox3, a key enzyme in the synthesis of crotonyl-CoA metabolism). The results of crotonylation modificaomics also showed that knockdown of Nrp1 in distal renal tubule cells significantly increased the levels of crotonylation modification of Cox4i1 (the key electron transport chain enzyme of oxidative phosphorylation), Immt (mitochondrial inner membrane protein), and Mdh1 (malate dehydrogenase) at the K29, K312, and K248 sites. Nrp1 can inhibit the aerobic metabolism of distal renal tubular epithelial cells (TECs) by reducing the crotonylation level of Cox4i1, thereby affecting mitochondrial function and aggravating renal injury (Li et al., 2024a).

Deletion of ACSS2 (acyl-CoA synthetase short chain family member 2) could influence H3K9cr level and thus improve kidney fibrosis. Studies have shown that whether *in vivo* or *in vitro*, H3K9cr level is significantly associated with IL-1β production. Tubular cell-derived IL-1β could stimulate M1 macrophage polarization to build a microinflammatory environment, which continues promoting persistent inflammation to damage tubular cells. Simultaneously, tubular cell-derived IL-1β could induce senescence of TECs. Furthermore, pharmacologic inhibition of ACSS2 can suppress H3K9cr-mediated IL-1β expression, which thereby alleviate IL-1β-dependent macrophage activation and tubular cell senescence to delay renal fibrosis. Thus, ACSS2 inhibitors may be a promising drug target for modifying histone crotonylation (Li et al., 2024b).

### 4.2 Neurological disease

Depression is one of the most common mental diseases in modern society, affecting the normal life of more than 300 million people worldwide. The development of depression is influenced by both genetic and environmental factors. Evidence also indicates the involvement of post transcription modification in the development of depression (Covington et al., 2011; Hobara et al., 2010). It has been found that the expression levels of histone crotonylation hydrolase and transcription inhibitor, CDYL, are significantly increased in the prelimbic cortex (PL) in a mouse model of stress-induced depression (Liu et al., 2019). Overexpression of CDYL in PL can increase the susceptibility of mice to depression, while knockdown of CDYL can reduce the susceptibility of mice to depression (Liu et al., 2019). CDYL affects structural synaptic plasticity by inhibiting the expression of the neuropeptide VGF mainly through the dual effect of histone crotonylation and H3K27 trimethylation on the VGF promoter. CDYL-VGF axis inhibits the structural synaptic plasticity of medial prefrontal cortex (mPFC), eventually leading to behavioral changes in susceptible individuals.

Alzheimer’s disease (AD), a degenerative disease of the central nervous system, is characterized by abnormal intracellular deposition of amyloid-beta (Aβ). Nuclear Paraspeckle Assembly Transcript 1 (NEAT1) exhibits repressed expression in the early stage of AD and its downregulation declines neuroglial cell mediating Aβ clearance via inhibiting expression of endocytosis-related genes. NEAT1 is associated with P300/CBP complex and silencing NEAT1 expression not only downregulated H3K27ac but also upregulated H3K27cr level, which might be caused by the NEAT1-mediated decrease of acetyl-CoA generation (Wang et al., 2019). Another study revealed that knockdown of NEAT1 can promot the enrichment of H3K27cr at the promoters of autophagy-related genes Beclin1, Atg3, and Atg5, and inhibits the transcription of autophagy-related genes through histone modification, thereby attenuating the autophagy function of neuroglial cells (Wang et al., 2022). Thus, NEAT1 may provide a potential therapeutic target for AD intervention.

Crotonylation modification also occurs in macrophages, sensory neurons, and satellite glial cells of trigeminal ganglia (TG), neurons, astrocytes, and microglia of the medulla oblongata. Peripheral nerve injury contributed to crotonylation level upregulation in macrophages but downregulation in sensory neurons in TG. Exogenously increased crotonyl-CoA could significantly upregulate the expression of inflammatory cytokines and chemokines. Decreasing crotonylation by inhibiting its writer p300 reduced pain and the production of multiple inflammatory mediators (Zou et al., 2022).

### 4.3 Infectious diseases

Human immunodeficiency virus (HIV) is a retrovirus which causes a multisystemic disease called acquired immunodeficiency syndrome (AIDS). Latent HIV reservoirs in the host are established very early in viral infection (Dandekar, 2007; Lackner et al., 2012; Somsouk et al., 2015; Hirao et al., 2014; Whitney et al., 2014). The stable viral reservoir in HIV patients is the main obstacle for the eradication of HIV, and the epigenetic regulation of histones N-terminus on HIV long terminal repeat (LTR) is the key to establishing the HIV viral reservoir (Margolis et al., 2016; Hakre et al., 2011). ASSC2 is a crotonyltransferase, which promotes the histone lysine crotonylation modification of HIV LTR, leading to reactivate the latent HIV (Jiang et al., 2018). Inhibitors of ACSS2 can inhibit viral replication and reactivation of latent HIV. Thus, inhibition of ACSS2 is expected to be a potential target for HIV eradication.

In addition, it was found that wogonin (a flavone isolated from Scutellaria baicalensis Georgi) could inhibit the reactivation of latent HIV-1 by inhibiting the expression of histone acetyltransferase p300 and reducing the crotonylation of histone H3/H4 in the HIV-1 promoter region. Wogonin is a novel latency-promoting agent (LPA) that can inhibit HIV-1 transcription by HIV-1 epigenetic silencing, which could bear promising significance for future applications of HIV-1 functional cure (Zhang et al., 2023).

*Brucella* is an intracellular parasitic bacterium that can cause chronic diseases in both humans and livestock. Type IV secretion systems (T4SS), as their important virulence factors, can secrete a variety of effector proteins. BspF (an effector member of T4SS) was detected with one crotonylation site. VirJ is a *Brucella* virulence factor involved in the T4SS secreted substrates, which undergoes two different types of PTMs, namely, 2-hydroxyisobutyrylation and crotonylation (Zhang et al., 2022a). Some proteins related to fungal pathogenicity were also found to be targets of crotonylation. Crotonylation modification sites were detected in *Trichophyton rubrum*, *Candida albicans*, and *Cryptococcus deneoformans* (Xu et al., 2022; Zhou et al., 2021; Li et al., 2021a).

### 4.4 Cancers

In recent years, the rise of epigenetics has helped us understand the pathogenesis of tumors. In addition to gene mutation, epigenetic regulation, especially abnormal protein modification, contributes to the development of tumors. Studies have shown that the levels of crotonylation are decreased in liver cancer, gastric cancer and renal cancer, while increased in thyroid, esophageal, colon, pancreatic and lung cancers (Wan et al., 2019b; Zhang et al., 2022b; Cai et al., 2021). The crotonylation in prostate tumors is significantly reduced, and expression levels of crotonylation proteins are positively correlated with tumor grade. Induction of histone crotonylation with bromodomain-containing protein 4 (BRD4) inhibitors can inhibit the proliferation and migration of prostate cancer cells, suggesting histone crotonylation can serve as biomarker for the diagnosis of prostate cancer and a therapeutic target for the treatment (Xu et al., 2021).

Histone H3K18 crotonylation modification is abnormally enriched in the transcription start site (TSS) of the small intestinal epithelium, especially in the crypt portion of the small intestine and colon. KEGG pathway analysis of crotonylation-modified genes in the TSS region reveals an enrichment in colorectal cancer (CRC)-related signaling pathways, indicating that abnormal histone crotonylation modification may be linked to CRC (Fellows et al., 2018). In addition, K420 crotonylation of ENO1 (α enolase) promotes the proliferation, migration and invasion of CRC cells by enhancing the enzyme activity of ENO1 and regulating the expression of tumor-related gene (Hou et al., 2021). It was found that H3K27cr levels were higher in CRC tissues than in adjacent tissues, and H3K27cr increased with CRC malignancy. Long noncoding RNA (lncRNA) binds and recruits crotonylation-modifying enzymes to specific genome loci. Overexpression of lncRNA LINC00922 prevents SIRT3 recruitment to the ETS1 promoter and then increases ETS1 transcription by increasing H3K27cr level in this region, ultimately promoting cancer metastasis (Liao et al., 2023).

Crotonylation also affects cervical cancer. For example, interfering the expression of p300 downregulates the levels of crotonylation modification and expression levels of HNRNPA1 protein. Treatment of Hela cells with crotonate can restore the crotonylation modification and increase expression levels of HNRNPA1 protein and proliferation of Hela cells (Han et al., 2020). Other proteins, such as SIRT1, SIRT2 and SIRT3, also regulate cervical cancer (Li et al., 2021b; Kuhlmann et al., 2017; Xu et al., 2020), but the mechanism of crotonylation modification in the regulation of these proteins has not been confirmed yet.

The YEATS domain plays an important regulatory role in leukemia. Inhibition of this domain decreases the transcription of oncogenes in leukemia. Because the affinity of YEATS domain to crotonylation sites is higher than that of other acylation sites, the epigenetic impact of the YEATS domain on leukemia may be partly due to its interaction with histone crotonylation. As one of the important domains to recognize crotonylation modification, YEATS domain is associated with the progression of various malignant tumors (Zeng et al., 2021; Han et al., 2022). At present, a variety of inhibitors targeting the binding of YEATS domain have been applied in the field of malignant tumor therapy (Listunov et al., 2021; Ma et al., 2021).

Glioblastoma (GBM) displays an immune suppressive microenvironment that dampens T cell infiltration, activation, and function, also limiting the efficacy of immunotherapy. It was found that glioblastoma stem cells (GSC) reprogram lysine catabolism, leading to accumulation of intracellular crotonyl-CoA and histone H4 lysine crotonylation. In the nucleus, glutaryl-CoA dehydrogenase (GCDH) interacts with the crotonyltransferase CBP to promote histone lysine crotonylation. Crotonylation derived from reprogrammed lysine catabolism serves as competitive epigenetic machinery to limit immunogenic transposable elements (TEs) through affecting H3K27ac and H3K9me3, which affects interferon signaling and CD8^+^ T cell infiltration, ultimately promoting tumor growth (Yuan et al., 2023).

Recently, it has been demonstrated that GCDH can inhibit the development, progression and metastasis of hepatocellular carcinoma (HCC). GCDH alters the cellular redox state through the crotonylation-induced pentose phosphate pathway (PPP) and glycolysis, leading to HCC cells senescence, which further induces infiltration of immune cells to form an anti-tumor immune microenvironment (Lao et al., 2024). The results suggest that increasing GCDH expression may be an effective strategy to improve the therapeutic outcome of HCC patients, and the expression level of GCDH may serve as an important indicator of the HCC clinical prognosis.

### 4.5 Cardiovascular disease

Posttranslational modifications of protein are critically involved in gene expression and regulate pathophysiologic processes such as cardiovascular diseases. In human newborns or children, mutations in the ECHS1 (Short-chain enoyl-CoA-hydratase) gene lead to cardiomyopathies, such as hypertrophic cardiomyopathy (Yamada et al., 2015). It was shown that ECHS1 regulates the level of crotonyl-CoA, which can regulate gene expression by histone crotonylation. H3K18cr and H2BK12cr levels are increased in human hypertrophic cardiomyopathy. In murine hypertrophic hearts, H3K18cr and H2BK12cr are remarkedly repressed by ECHS1 overexpression. By contrast, crotonyl-CoA supplement with crotonate increased histone crotonylation in cardiomyocytes, which was enhanced by Echs1 knockdown, suggesting that ECHS1 inhibits histone crotonylation (Tang et al., 2021). Therefore, histone crotonylation may serve as a therapeutic target for children with ECHS1 mutations and patients with hypertrophic cardiomyopathy.

Proteomic analysis demonstrated that acute myocardial ischemia-reperfusion (I/R) injury triggers extensive crotonylation, specifically of proteins associated with cardiomyocyte contractility, including mitochondrial, cytoskeletal, sarcoplasmic reticulum, and gap junction proteins. Crotonylation of IDH3a (isocitrate dehydrogenase 3 [NAD+] alpha) at K199 and cytoskeletal protein TPM1 (tropomyosin alpha-1 chain) at K28/29 can protect cardiomyocyte from apoptosis by inhibiting BNIP3 (Bcl-2 adenovirus E18 19-kDa-interacting protein 3)-mediated mitosis or cytoskeleton structure rearrangement. Sodium crotonate (NaCr)-enhanced general lysine crotonylation preserves myocardial function following I/R injury (Cai et al., 2022). Thus, modulating specific and co-ordinated crotonylation represents a potential novel cardiomyocyte-targeted strategy for therapeutic intervention to improve ischemic heart disease outcomes.

A recent study shows crotonylation of non-histone NAE1 (NEDD8 [neural precursor cell expressed developmentally downregulated protein 8]-activating enzyme E1 regulatory subunit) is involved in the regulation of cardiac hypertrophy. GSN (gelsolin) is a direct target of NAE1. K238 crotonylation of NAE1 can promot GSN neddylation (a posttranslational modification), which enhances stability and expression of the GSN protein. Then increased GSN promotes actin-severing activity, leading to adverse cytoskeletal remodeling and cardiac hypertrophy (Ju et al., 2024).

### 4.6 Metabolic disease

Browning of white fat is considered to be an important tool to counteract obesity. SIRT3 was identified as a decrotonylation and deacetylation modification enzyme that regulates dihydrolipoyl dehydrogenase (DLD). DLD promotes browning of white adipocytes by activating mitochondrial function through crotonylation modification and the RAS/ERK pathway, providing a theoretical basis for the control and treatment of obesity (Liu et al., 2024).

The incidence of type 2 diabetes mellitus (T2DM) has increased dramatically, and T2DM has become a global health concern. Enteromorpha prolifera oligosaccharide (EPO) possesses the excellent ability of anti-oxidative, anti-inflammatory, and anti-diabetic. Recent studies have shown that EPO is able to alter the crotonylation level of XPO1, which can reduce cellular oxidative damage and retard the aging process. Moreover, EPO could affect glucose metabolism by reducing crotonylation of HSPA8 and regulating the AKT1 related pathway to achieve anti-diabetic effect (Shan et al., 2024).

## 5 Conclusion and perspective

Crotonylation is a novel PTM present in a variety of proteins in prokaryotes and eukaryotes (Sun et al., 2020; Wang et al., 2020; Bao et al., 2020; Zhang et al., 2020). Crotonylation modification is involved in regulation of various biological functions from gene expression to protein stability. The diversity of modification sites enables crotonylation not only on histones, but also in other non-histone lysine residues. The crotonylation writers, erasers, and readers are generally shared with other PTMs, but it is still unclear whether specific enzymes for crotonylation exist. The underlying mechanisms of crotonylation and its dynamic relationship with other acylations need to be further explored.

Similar to protein lysine acylation modifications, such as acetylation and succinylation, crotonylation can be regulated by enzymatic or non-enzymatic reaction. Cr-CoA is considered to be the donor of crotonylation modification and its short-chain fatty acid synthesis is derived from crotonate. The concentration of intracellular crotonate or Cr-CoA can affect the levels of crotonylation modification (Sabari et al., 2015). Therefore exploring the factors that influence crotonyl-CoA levels is also a promising field.

In addition, the histone and non-histone proteins crotonylation associated diseases were discussed deeply here. Crotonylation may exert diverse regulatory effects on disease development depending on the specific tissues, organs, cells, and cellular microenvironments involved. The clarification of the crotonylation mechanism has the potential to provide practical therapeutic modalities for the treatment of several diseases, including those that are difficult to cure, such as kidney diseases and cancer. It will be interesting to determine how crotonylation mechanistically regulates their function in the near future.
